# Establishment of a *Mycoplasma hyorhinis* challenge model in 5-week-old piglets

**DOI:** 10.3389/fmicb.2023.1209119

**Published:** 2023-08-04

**Authors:** Dorottya Földi, Zsófia Eszter Nagy, Nikolett Belecz, Levente Szeredi, József Földi, Anna Kollár, Miklós Tenk, Zsuzsa Kreizinger, Miklós Gyuranecz

**Affiliations:** ^1^Veterinary Medical Research Institute, Budapest, Hungary; ^2^National Laboratory of Infectious Animal Diseases, Antimicrobial Resistance, Veterinary Public Health and Food Chain Safety, Budapest, Hungary; ^3^Department of Microbiology and Infectious Diseases, University of Veterinary Medicine, Budapest, Hungary; ^4^Euvet Bt., Gödöllo, Hungary; ^5^MolliScience Kft., Biatorbágy, Hungary

**Keywords:** challenge, ELISA, infection, *Mycoplasma hyorhinis*, PCR, pig

## Abstract

**Introduction:**

*Mycoplasma hyorhinis* is an emerging swine pathogen with high prevalence worldwide. The main lesions caused are arthritis and polyserositis, and the clinical manifestation of the disease may result in significant economic losses due to decreased weight gain and enhanced medical costs. We aimed to compare two challenge routes to induce *M. hyorhinis* infection using the same clinical isolate.

**Methods:**

Five-week-old, Choice hybrid pigs were inoculated on 2 consecutive days by intravenous route (Group IV-IV) or by intravenous and intraperitoneal routes (Group IV-IP). Mock-infected animals were used as control (control group). After the challenge, the clinical signs were recorded for 28 days, after which the animals were euthanized. Gross pathological and histopathological examinations, PCR detection, isolation, and genotyping of the re-isolated *Mycoplasma* sp. and culture of bacteria other than *Mycoplasma* sp. were carried out. The ELISA test was used to detect anti-*M. hyorhinis* immunoglobulins in the sera of all animals.

**Results:**

Pericarditis and polyarthritis were observed in both challenge groups; however, the serositis was more severe in Group IV-IV. Statistically significant differences were detected between the challenged groups and the control group regarding the average daily weight gain, pathological scores, and ELISA titers. Additionally, histopathological scores in Group IV-IV differed significantly from the scores in the control group. All re-isolated strains were the same or a close genetic variant of the original challenge strain.

**Discussion:**

Our results indicate that both challenge routes are suitable for modeling the disease. However, due to the evoked more severe pathological lesions and the application being similar to the hypothesized natural route of infection in Group IV-IV, the two-dose intravenous challenge is recommended by the authors to induce serositis and arthritis associated with *M. hyorhinis* infection.

## Introduction

*Mycoplasma hyorhinis* is an emerging pathogenic bacterium of swine, the distribution of which is considered to be worldwide with a high estimated prevalence (Pieters and Maes, 2019; Roos et al., 2019). *M. hyorhinis* colonizes the upper respiratory tract and tonsil of sows, which are asymptomatic carriers of the bacterium. Piglets get infected directly from the nasal secretions of sows, and later from each other, especially after weaning (Clavijo et al., 2017). Clinical signs usually appear between 3 and 10 weeks of age. Although the susceptibility to the infection decreases after this age, pigs can get infected even up to 16 weeks of age (Martinson et al., 2017). The pathomechanism of systemic spread is still not fully understood. Predisposing factors such as inadequate housing conditions or weaning and decreasing maternal antibodies at ~3 weeks of age can all contribute to the disease (Clavijo et al., 2019).

The first clinical signs appear 3–10 days post-exposure and include fever and lethargy (Gomes Neto, 2012). Later, coughing, labored breathing, and dyspnea can appear due to serofibrinous pleuritis, pericarditis, and peritonitis. Additionally, arthritis with swollen joints and lameness can be observed in pigs (Barden and Decker, 1971). Rarely, *M. hyorhinis* infection causes otitis (Morita et al., 1995), conjunctivitis (Resende et al., 2019), and meningitis (Bünger et al., 2020). Affected pigs show growth retardation, which can be evident even 5 months after infection (Barden and Decker, 1971). As a secondary pathogen, *M. hyorhinis* can aggravate the clinical signs of other infections such as porcine circovirus 2-associated diseases and enzootic pneumonia and is also an important pathogen in the porcine respiratory disease complex (Pieters and Maes, 2019). Decreased weight gain and the cost of medical treatments result in significant economic losses. As no commercial vaccine is available in Europe, prevention mainly relies on decreasing predisposing factors; however, metaphylactic antibiotic treatment is often required.

There are some *M. hyorhinis* challenge models in the literature suggesting different inoculation routes (Lin et al., 2006; Gomes Neto et al., 2014; Lee et al., 2018; Fourour et al., 2019; Merodio et al., 2021). However, not all published models are suitable for vaccine efficacy studies, because with some of the suggested challenge routes not all typical lesions can be induced (Martinson et al., 2018a).

We aimed to compare the effects of experimental infections of two distinct inoculation routes with the same virulent *M. hyorhinis* strain by studying the clinical signs, immune response, and macroscopic and microscopic alterations. Accordingly, the examinations also aimed to establish a challenge model for future vaccine efficacy studies.

## Materials and methods

### Challenge material

The *M. hyorhinis* isolate used during this study was isolated from the pericardium of a pig affected by pericarditis, originating from Hungary in 2019. The initial isolation was carried out using the filter cloning technique in Mycoplasma Experience Medium (Mycoplasma Experience Ltd., Bletchingley, UK), and the isolate was identified by the partial sequencing of the 16S-23S rRNA intergenic spacer region, using *Mycoplasma* genus-specific primers (Lauerman et al., 1995); the sequencing was performed on an ABI Prism 3100 automated DNA sequencer (Applied Biosystems, Waltham, MA, USA), followed by sequence analysis and BLASTN search (https://blast.ncbi.nlm.nih.gov/Blast.cgi). The challenge material was prepared freshly for each challenge day by inoculating the fourth passage of the isolate 48 h prior challenge to Mycoplasma Experience Medium (Mycoplasma Experience) and incubating at 37°C. The color-changed broth was directly used for the challenge. The viable cell count determination was carried out on the day of the challenge. The number of color-changing units (CCU/ml) was calculated by broth micro-dilution from the highest dilution showing color change (red to yellow shift; Hannan, 2000).

###  Experimental animals

Sixteen, 4-week-old Choice hybrid piglets were transported to the animal house of the Veterinary Medical Research Institute 6 days prior infection. The animals were obtained from a farm with low *M. hyorhinis* prevalence and high health status (free from brucellosis, leptospirosis, Aujeszky's disease, porcine reproductive and respiratory syndrome, swine dysentery, atrophic rhinitis, *Actinobacillus pleuropneumoniae, Mycoplasma hyopneumoniae*, lice, and mange). The *M. hyorhinis*-free status of the piglets was checked before the challenge by real-time PCR testing and *Mycoplasma* culture of nasal swabs.

Upon arrival, the animals were weighed and randomly divided into three groups with similar average weights. The animals were of mixed gender, which was not a factor when the groups were formed. The groups were housed in separate pens of 6 m^2^ each. Humidity and air quality were regulated by the built-in ventilation system, and the age-appropriate temperatures were provided by heating panels. Feed and water were provided *ad libitum*. The experiment was approved by the National Scientific Ethical Committee on Animal Experimentation under reference number: PE/EA/746-7/2021.

###  Challenge routes

Group IV-IV (*n* = 6) was inoculated by intravenous (IV) route on 0 and 1 day post-infection (0 DPI, 1 DPI) with 10 ml challenge material. Group IV-IP (*n* = 6) was challenged IV on 0 DPI with a 10-ml challenge material and intraperitoneal (IP) route on 1 DPI with a 20-ml challenge material. For the IV inoculation, the external jugular vein was used, while for the IP route, the challenge material was injected into the abdominal cavity in the left lower abdominal quadrant. On 0 DPI, the viable cell count of the challenge strain was 4.6 × 10^5^ CCU/ml, while on 1 DPI, it was 1.2 × 10^6^ CCU/ml. The total challenge dose was 1.66 × 10^7^ CCU/pig and 2.86 × 10^7^ CCU/pig in Group IV-IV and IV-IP, respectively. The controls (*n* = 4) were inoculated by IV route on 0 DPI. Two of these animals were inoculated by the IV route and the remaining two pigs by the IP route on 1 DPI. Animals in the control group received only sterile liquid media in the same volume as the challenged groups.

###  Clinical observation

The animals were observed daily from settlement until the end of the study, at 28 DPI. Clinical signs of arthritis [swollen joints (mostly detected visually, complemented with palpation) and lameness] and respiratory disease (coughing or labored breath) were recorded. Body temperatures were measured daily from 2 days prior challenge. Body weight measurement, blood, and nasal swab sampling were carried out twice a week. Cotton swabs were used for collecting nasal swabs (Swab in tube Alum + Cotton, Deltalab S.L., Barcelona, Spain), and blood was collected from the external jugular vein. The schedule of events is summarized in Table 1. Average daily weight gain (ADWG) was calculated by subtracting the weight measured at arrival (6 days prior challenge, −6 DPI) from the weight measured at 27 DPI and dividing it by the number of days past (*n* = 33).

**Table 1:** Schedule of events, challenge routes, and doses.

**Time**	**Event**	**Challenge dose**
−6 DPI	Arrival of 16 four-week-old piglets Body weight measurement	
−2 DPI	Blood sampling Collection of nasal swabs for PCR and *Mycoplasma* isolation Body temperature measurement	
−1 DPI	Body temperature measurement	
0 DPI	IV challenge of all groups	10 ml 10^6^ CCU/ml challenge material (Groups IV-IV and IV-IP) or 10 ml sterile broth (control group)
1 DPI	IV challenge of group IV-IV and two animals from control group IP challenge of group IV-IP and two animals from control group	IV: 10 ml 10^6^ CCU/ml challenge material (group IV-IV) or 10 ml sterile broth (control group) IP: 20 ml 10^6^ CCU/ml challenge material (group IV-IP) or 20 ml sterile broth (control group)
0–27 DPI	Daily: - Body temperature measurement - Clinical observations Twice a week: - Body weight measurement - Collection of nasal swabs for PCR and *Mycoplasma* isolation - Blood sampling	
28 DPI	Euthanasia (after electrical stunning exsanguination was performed) Pathological examination Sample collection for PCR, histopathology, and bacteriology	

IV, intravenous; IP, intraperitoneal; CCU, color changing unit; DPI, days post-infection.

###  Isolation, DNA extraction, and PCR

Nasal swabs for *Mycoplasma* isolation and PCR were taken twice a week from all animals throughout the study. Separate swab samples for *Mycoplasma* isolation and PCR were collected during necropsy as well (see below). For *Mycoplasma* isolation, swabs were cut into Mycoplasma liquid media (Mycoplasma Experience Ltd.), and vortex mixed to aid the release of bacteria into the media, which was then filtered using 0.45 μm pore size filters and incubated at 37°C until color change.

DNA extraction from the swabs and color-changed broths was performed using a ReliaPrep gDNA Tissue Miniprep System (Promega Inc., Madison, USA) according to the manufacturer's instructions. For the *M. hyorhinis* species-specific real-time PCR, previously published (Resende et al., 2019) primers targeting the 16S rRNA gene were optimized. Primer and probe sequences were the following: Forward primer 5′- CGT ACC TAA CCT ACC TTT AAG−3′, Reverse primer 5′- TAA TGT TCC GCA CCC C−3′, Probe 5′- FAM-CCG GAT ATA GTT ATT TAT CGC ATG AG-BHQ−3′. The PCR was performed using a Bio-Rad C1000 Touch™ Thermal Cycler, CFX96™ Real-Time System (Bio-Rad Laboratories Inc., USA). The PCR master mix consisted of 6 μl 2 × qPCRBIO Probe Mix No-ROX (PCR Biosystems Ltd., UK), 0.4 μl of each primer (10 μM), 0.2 μl probe, and 2 μl DNA in the final volume of 12 μl. PCR conditions were the following: 95°C for 2 min, 45 cycles of 95°C for 5 s, and 60°C for 20 s. In order to test the sensitivity of the developed assays, 10-fold dilutions of the DNA of the type strain (NCTC 10130) were used in the range of 10^6^-10^0^ template copy number/μl. Template copy number was calculated with the help of an online tool (http://cels.uri.edu/gsc/cndna.html) by measuring the concentration of DNA of pure *M. hyorhinis* culture using a Nanodrop 2000 Spectrophotometer (Thermo Fisher Scientific Inc., USA). The lowest DNA concentration giving a specific signal was considered the detection limit of the assay. The specificity was tested by including *M. hyopneumoniae, Mycoplasma hyosynoviae*, and *Mycoplasma flocculare* in the analyses.

Necropsy samples were also tested for the presence of *M. hyopneumoniae* (Wu et al., 2019) and *M. hyosynoviae* (Martinson et al., 2018a) using PCR. *M. hyorhinis* positive isolates were genetically characterized by multi-locus sequence typing (MLST: used as a costly but robust genotyping system) and multiple-locus variable-number tandem-repeat analysis (MLVA: used as a rapid and cheap genotyping system with high-resolution), according to previously published assays (Földi et al., 2020).

###  Gross pathological examination

Joints of the carpus, elbow, tarsus, and stifle on both sides were opened and examined for signs of arthritis. The thoracic and abdominal cavities (pleura, pericardium, and peritoneum) were checked for serositis. Body condition, skin, subcutaneous tissues, musculoskeletal system, eyes and conjunctiva, nasal, and oral cavity, trachea, lungs, heart, lymph nodes, gastrointestinal system, liver, spleen, kidney, and brain were also checked for lesions. The scoring system of the gross pathological examination is detailed in Supplementary Table 1. Lesions of joints and serosa were scored to reflect severity based on previously described criteria (Martinson et al., 2018b). Total scores were calculated by summarizing all organ scores.

Swab samples for bacterial culture, *M. hyorhinis* isolation, and PCR were taken from the conjunctiva, lung, serosa, the four examined joints, and the brain. Joints on both sides were sampled with the same swab. During necropsy, viscose swabs were used (Swab in tube PS + Viscose, Deltalab).

###  Histological examination

Samples for histopathology were collected from the conjunctiva, choana, tonsilla, trachea, lungs (seven lobes), pericardium, heart, mediastinal and mesenteric lymph nodes, liver, spleen, kidney, joints, and brain (cerebrum, cerebellum, and brain stem). Tissue samples were fixed in 10% formaldehyde and embedded in paraffin, and then, 4 μm thick sections were cut and stained with hematoxylin and eosin (H&E) and examined by light microscope. Given the limited number of examined animals in the present study, the establishment of a general scoring system was not possible. Therefore, lesions were categorized based on the comparison of the severity of the histopathological changes with each other. The criteria of the scoring system used in this study are detailed in Supplementary Table 2.

###  Bacteriology

The presence of bacterial pathogens other than *Mycoplasma* sp. was tested by culturing the necropsy samples on Columbia sheep blood agar (Biolab Inc., Hungary) and sheep blood agar supplemented with nicotinamide adenine dinucleotide (Sigma-Aldrich Co., USA) at the final concentration of 20 μg/ml. The agar plates were incubated in the presence of 5% CO_2_ at 37°C for 48 h.

###  Serology

Sera were tested in duplicates by an in-house ELISA, using an antigen prepared according to the sarcosyl assay described previously (Stipkovits et al., 1993). In brief, to prepare the antigen, six clinical isolates of *M. hyorhinis* were propagated (Supplementary Table 3). After the color changes, the isolates were mixed, washed, and treated with 0.5% sarcosyl. The protein content of the antigen was determined using a Coomassie (Bradford) Protein assay kit (Thermo Fisher Scientific Inc.) according to the manufacturer's instructions.

Furthermore, 96-well ELISA plates were coated with the antigen diluted to the concentration of 1.25 μg/ml in phosphate-buffered saline (PBS, pH 7.4). After blocking with 1% gelatin from cold water fish skin (Sigma-Aldrich Co.), each well was incubated with a serum sample diluted to 1:100 in PBS, followed by a horseradish peroxidase-conjugated rabbit anti-swine immunoglobulin (Dako A/S, Denmark) diluted to 0.125 μg/ml in PBS. The reaction was visualized with tetramethylbenzidine substrate (TMB, Diavet Ltd., Hungary), and the optical density of the solution was measured at 450 nm using a Multiscan FC reader (Thermo Fisher Scientific Inc.).

Blood samples were centrifuged after collection, and the sera were maintained at −70°C. Each serum sample was thawed only once. Each plate contained a negative control (mix of the sera of each control animal taken at 28 DPI from this study), a positive control (mix of the sera of each animal in Group IV-IV taken at 28 DPI from this study), and a background control, where PBS was measured instead of the serum sample. The mean OD value of the background control was subtracted from the mean OD values of the samples and the controls (Terato et al., 2016). Samples were considered positive when the OD values were higher than three times the mean OD values of the negative controls (Lardeux et al., 2016). The mean of all negative controls used in the ELISAs was used to calculate this value.

### Statistical analyses

Statistical analyses were accomplished using the R program (R Core Team, 2021). To compare the effect of the different challenge routes statistical analysis of the ADWG, pathological scores (separately for the joints, serosa of the pericardium, pleura, and peritoneum, and summary of scores), histopathological scores (separately for the joints, serosa of the pericardium, pleura, and peritoneum, and summary of scores), and ELISA results from the last sampling were performed. In the case of the pathological and histopathological scores, first, a Kruskal–Wallis non-parametric ANOVA test was carried out to determine whether the differences among the medians of the three study groups are statistically significant or not. If the results of the Kruskal–Wallis test were significant, Dunn's test was performed to determine exactly which groups are different by making pairwise comparisons between each group. Since multiple groups were considered at the same time, *p*-values were adjusted for multiple comparisons by the Bonferroni method. In the case of the ADWG and the ELISA results, instead of the non-parametric test, a one-way ANOVA followed by Tukey's multiple comparisons of means was performed after the normal distribution of the data was tested using the Shapiro–Wilk normality test.

## Results

###  Clinical observations

No clinical alterations were detected in the control group throughout the study. No body temperature higher than 40.3°C was recorded during the study. One pig in Group IV-IP had a body temperature higher than 40°C on 3 consecutive days (4–6 DPI, Supplementary Table 4). No respiratory signs were recorded in the challenge groups.

Swollen joints were detected as early as 6 DPI in Group IV-IP and 8 DPI in Group IV-IV. Typically, the first swollen joint was one of the tarsal joints. By 16 DPI, all pigs in Group IV-IP had at least one swollen joint, three out of six pigs had two swollen tarsi, and in one animal, carpal joints were also affected. In Group IV-IV, by 20 DPI, swollen tarsal joint was observed in four out of six pigs (one side only), and in one animal, both tarsi were affected, while no swollen joints were detected in one pig (Supplementary Table 5).

**Figure 1:**
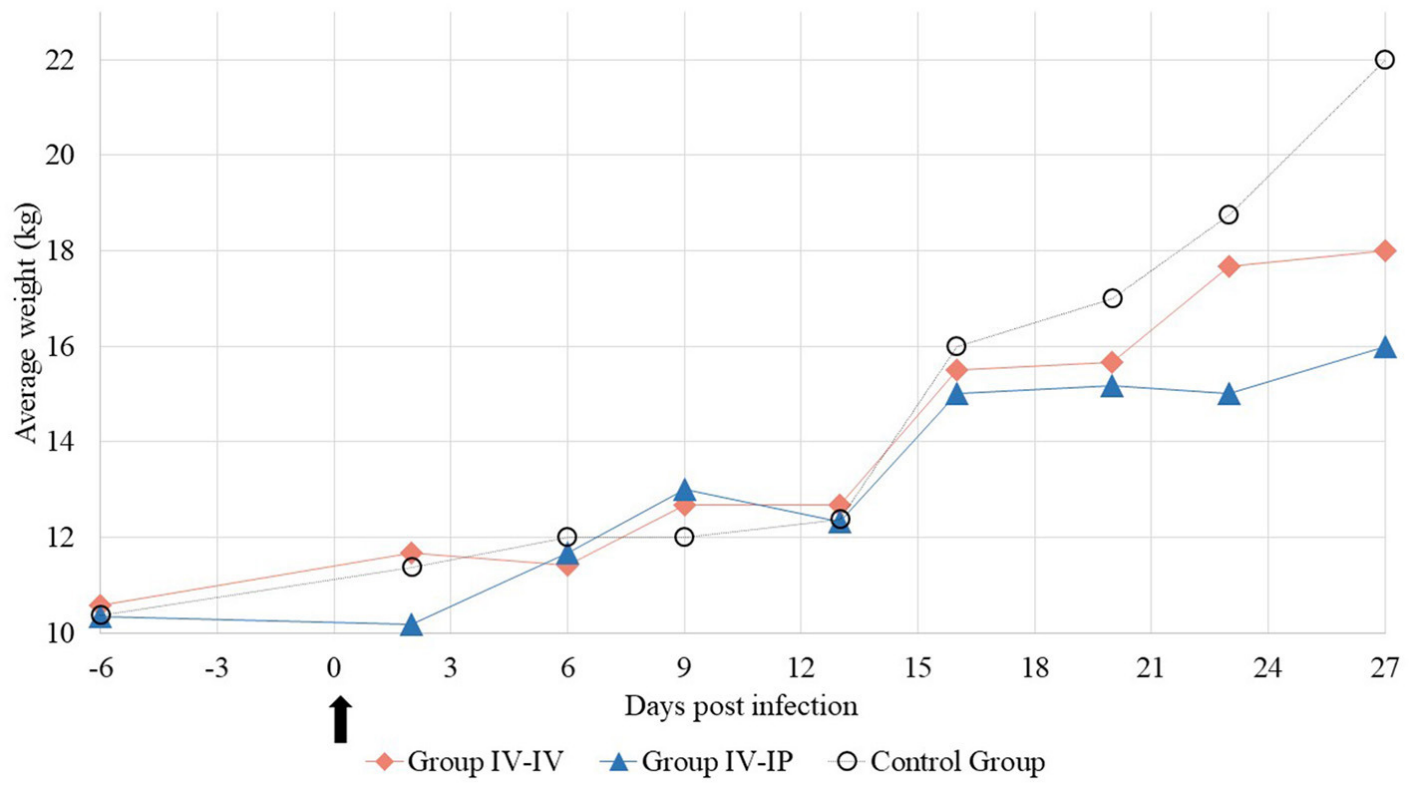
Average weight of the study groups at each sampling point. The arrow marks the first day of the challenge.

Weight gain dynamics of the different groups are shown in Figure 1 and detailed in Supplementary Table 6. The average starting weights of the groups were 10.5 kg (SD 1.2), 10.3 kg (SD 1.2), and 10.3 kg (SD 1.1) in Groups IV-IV, IV-IP, and control, while at the end of the study average body weights of the groups were 18.0, 16.0, and 22.0 kg, respectively. Mean ADWG was 223, 170, and 350 g in Groups IV-IV, IV-IP, and control, respectively. Significant differences in ADWG were detected between control and IV-IV groups (*p* = 0.05) and control and IV-IP groups (*p* < 0.01; Supplementary Data 1).

### *Mycoplasma* isolation, PCR, and bacteriology

The sensitivity of the reaction with the optimized primers was 10^1^ copies/reaction, and no cross-reactions were detected for *M. hyopneumoniae, M. hyosynoviae*, and *M. flocculare*.

Nasal swabs of all animals were negative for *M. hyorhinis* by PCR and isolation at the beginning of the study (2 days prior challenge). After the inoculation of the pigs, one sample from each challenged group was positive by isolation which was also positive by PCR either at the same time or at different sampling times. These animals remained PCR positive for 2–4 consecutive sampling points. Furthermore, two animals in Group IV-IV and one animal in Group IV-IP were PCR-positive as well at one sampling point. All nasal samples of the control group were negative by PCR and isolation for *M. hyorhinis* throughout the study (Supplementary Table 7).

Samples collected from the conjunctiva and meninx during necropsy were negative for the tested mycoplasmas in all animals, while one lung sample in Group IV-IV was positive for *M. hyorhinis* by PCR. Three samples from different serosa (pleura, pericardium, and peritoneum) were positive by PCR as well in Group IV-IV. A high number of joint samples were positive by PCR in both challenged groups. In Group IV-IV, two out of six stifle, four out of six elbow, five out of six tarsus, and four out of six carpus samples were positive for *M. hyorhinis* by PCR. However, in Group IV-IP, two out of six stifle, four out of six elbow, four out of six tarsus, and one out of six carpus samples were positive. All samples from the control group were negative for *M. hyorhinis* (Supplementary Table 8). *M. hyopneumoniae* or *M. hyosynoviae* were not detected in any samples collected during necropsy.

During the challenge study, two nasal isolates and isolates from six necropsy samples (tarsal, carpal, elbow, and stifle joints) were collected and their genotypes were first determined by MLVA. Two re-isolates in Group IV-IP differed from the challenge strain on one allele (MHR444; Supplementary Table 9). They were micro-variants due to within-host evolution. The sequence types of these two isolates, two other isolates from the same animals, and one isolate from Group IV-IV were also determined by MLST. All the re-isolated strains showed the same sequence type (ST) with MLST as the challenge strain (GenBank IDs: OR250721-OR250756). MLST and MLVA trees are shown in Supplementary Figure 1.

None of the cultures of the necropsy samples showed growth of pathogenic bacteria that could also be associated with the lesions, other than *M. hyorhinis*.

###  Gross pathological examination

**Figure 2:**
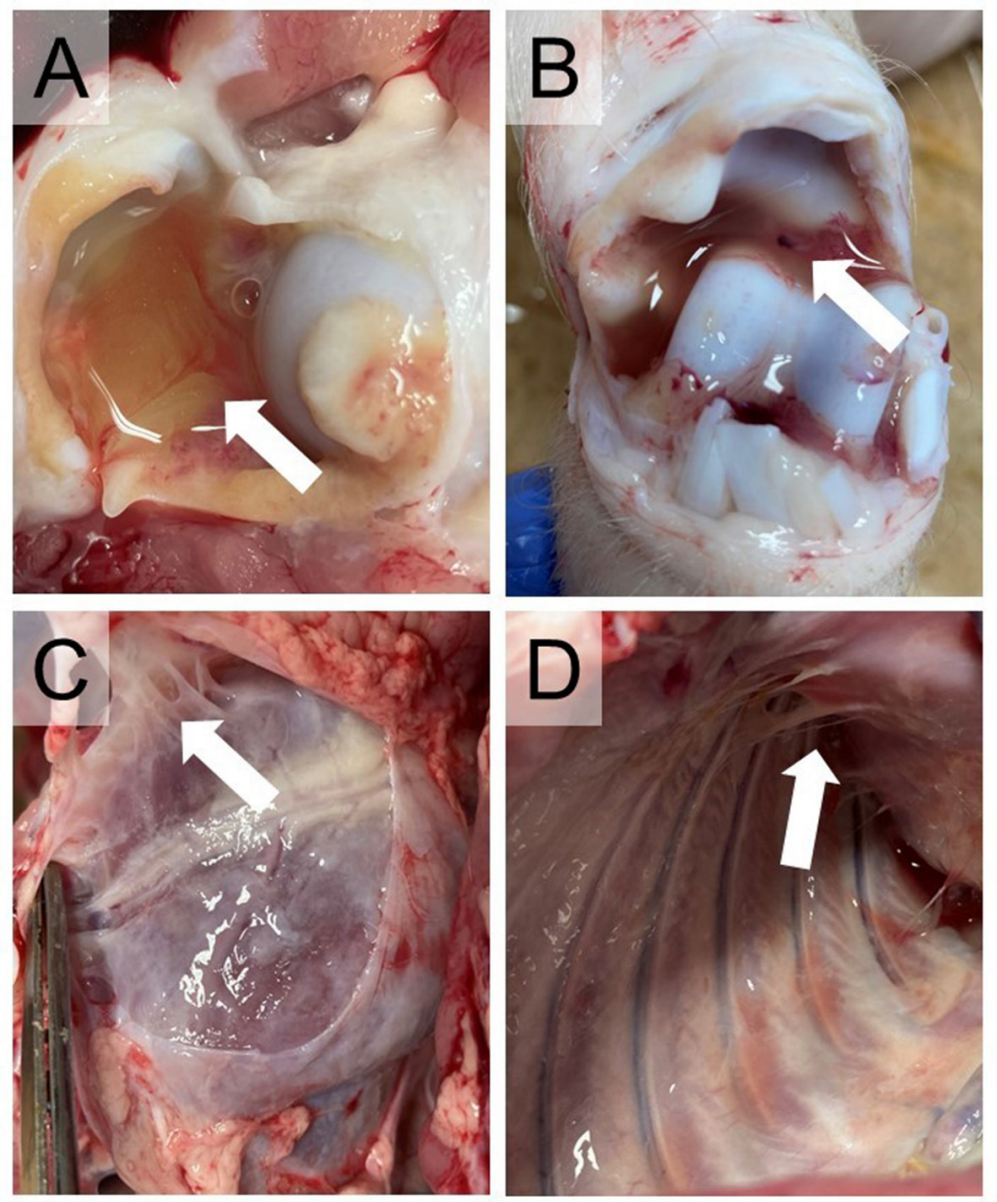
Typical lesions of *Mycoplasma hyorhinis* infection. **(A)** Joint with excess synovial fluid. **(B)** Serosanguinous synovial fluid. **(C)** Serofibrinous pericarditis. **(D)** Serofibrinous pleuritis.

Arthritis of at least one joint was observed in all pigs in the challenge groups. Mild-to-severe arthritis was found in all joints examined in one pig and in three joints examined in another pig in Group IV-IV. A single joint was affected in the remaining four animals in this group. Mild-to-severe arthritis was found in three, two, or one joints of two-two pigs in Group IV-IP. Arthritis manifested as serous or purulent inflammation (Figures 2A, B) and was detected most often in the tarsus (8/12) followed by the elbow (6/12), stifle (5/12), and carpus (4/12) on one or on both sides.

Diffuse, severe, chronic pericarditis presenting a large amount of connective tissue was detected in two animals in both challenge groups (Figure 2C). Additionally, mild or moderate chronic pleuritis presenting filaments of connective tissues were detected in two animals (Figure 2D) and mild chronic peritonitis presenting filaments of connective tissues occurred in one other animal in Group IV-IV.

**Figure 3:**
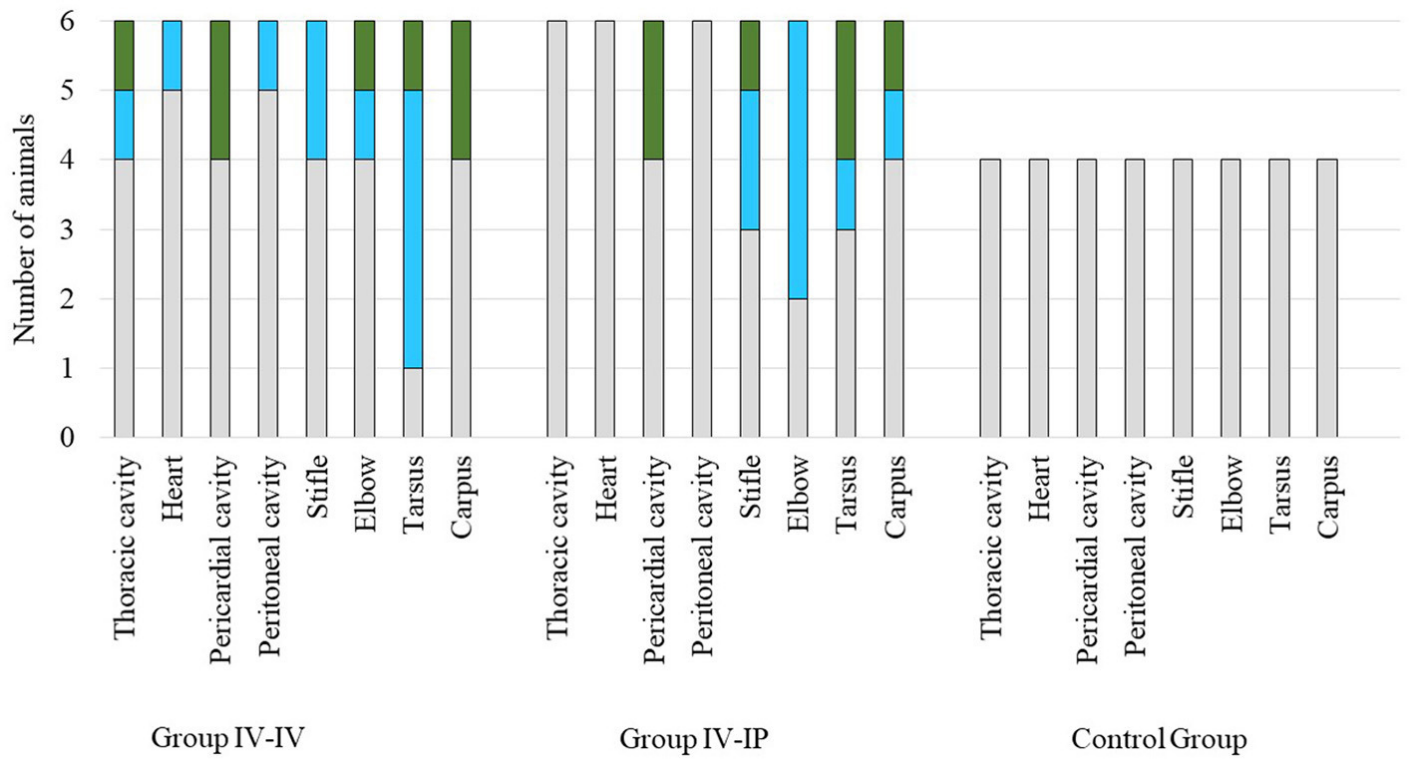
Scores of macroscopic lesions of the affected organs of the study groups. Organs were scored between 0 and 2 based on the severity of the lesion, except for the heart where score 1 was given in case of any lesion (Supplementary Table 1). Color codes in the charts: gray indicates the number of animals with a score of 0, blue indicates the number of animals with a score of 1, and green indicates the number of animals with a score of 2. The number of animals in each group is indicated on the *Y*-axis: the challenge groups consisted of six animals, while the control group involved four animals.

Macroscopic scores of lesions in the affected organs are demonstrated in Figure 3. No gross pathological alterations were found in the remaining organs examined. No gross pathological lesions were detected in any examined organs in the control group. Body condition in all groups was normal. Detailed pathological scores are given in Supplementary Table 1.

Significant differences in pathological scores were detected when scores of joint lesions and total scores of groups were compared. Pathological scores in both challenge groups differed significantly from the control group (*p* = 0.03 and *p* = 0.02 regarding joint lesions, *p* = 0.02 and *p* = 0.03 regarding total scores for Group IV-IV–control group and Group IV-IP–control group, respectively) but not from each other in both cases. No significant difference was found when scores of serosa lesions were compared (Supplementary Data 1).

###  Histological examination

**Figure 4:**
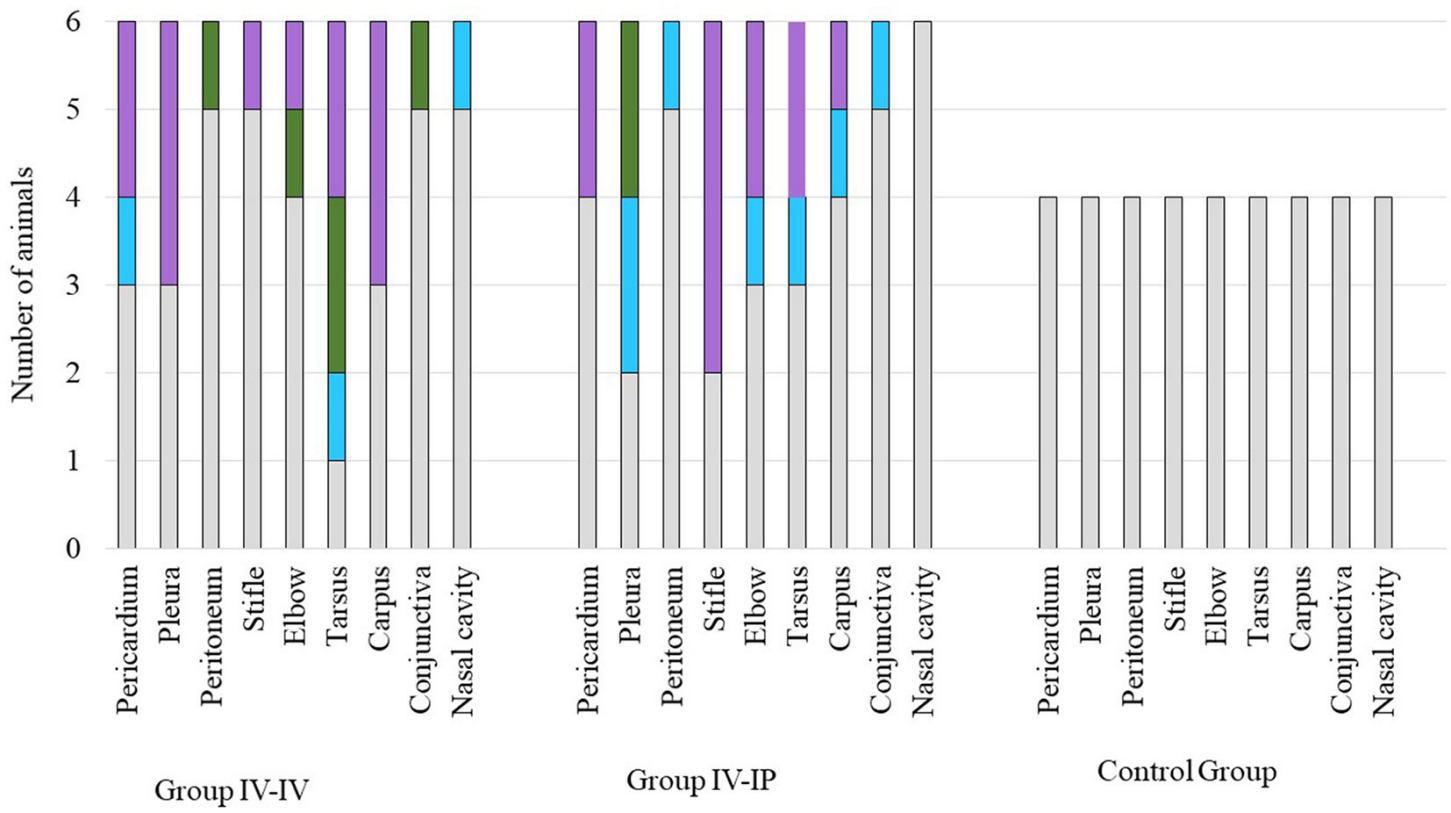
Scores of histopathologic lesions of the affected organs. Organs were scored between 0 and 3 based on the severity of the lesion. Color codes in the charts: gray indicates the number of animals with a score of 0 (no lesion), blue indicates the number of animals with a score of 1 (mild lesions), green indicates the number of animals with a score of 2 (moderate lesions), and lilac indicates the number of animals with a score of 3 (severe lesions). The number of animals in each group is indicated on the *Y*-axis: the challenge groups consisted of six animals, while the control group involved four animals.

**Figure 5:**
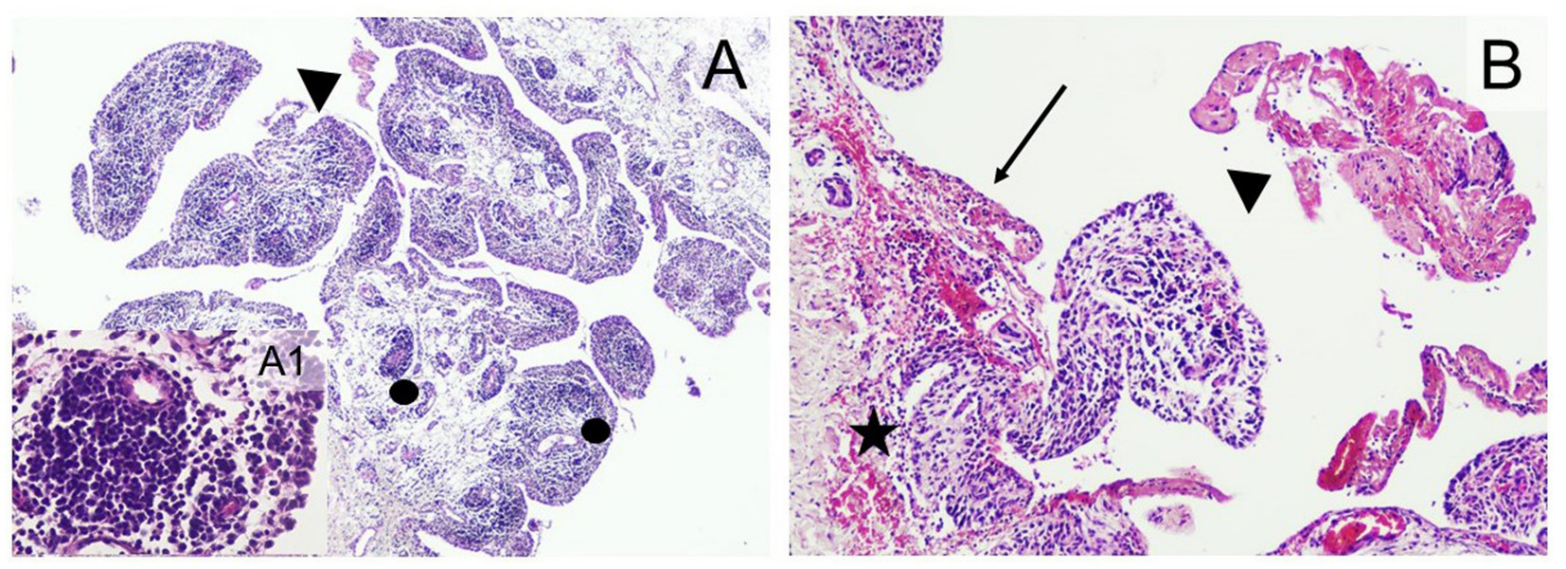
Typical histopathological changes in the joints of *Mycoplasma hyorhinis*-infected piglets. **(A)** Severe arthritis with the formation of perivascular lymphoid follicles (dots) and fibrin exudates in the joint cavity (arrowhead; 40×, H&E). A1: Perivascular follicle (400×; H&E). **(B)** erosive synovitis (arrow) associated with acute hemorrhages (star), frequent occurrence of fibrin exudates, blood cells, and neutrophil granulocytes in the joint cavity (arrowhead; 100×; H&E).

**Figure 6:**
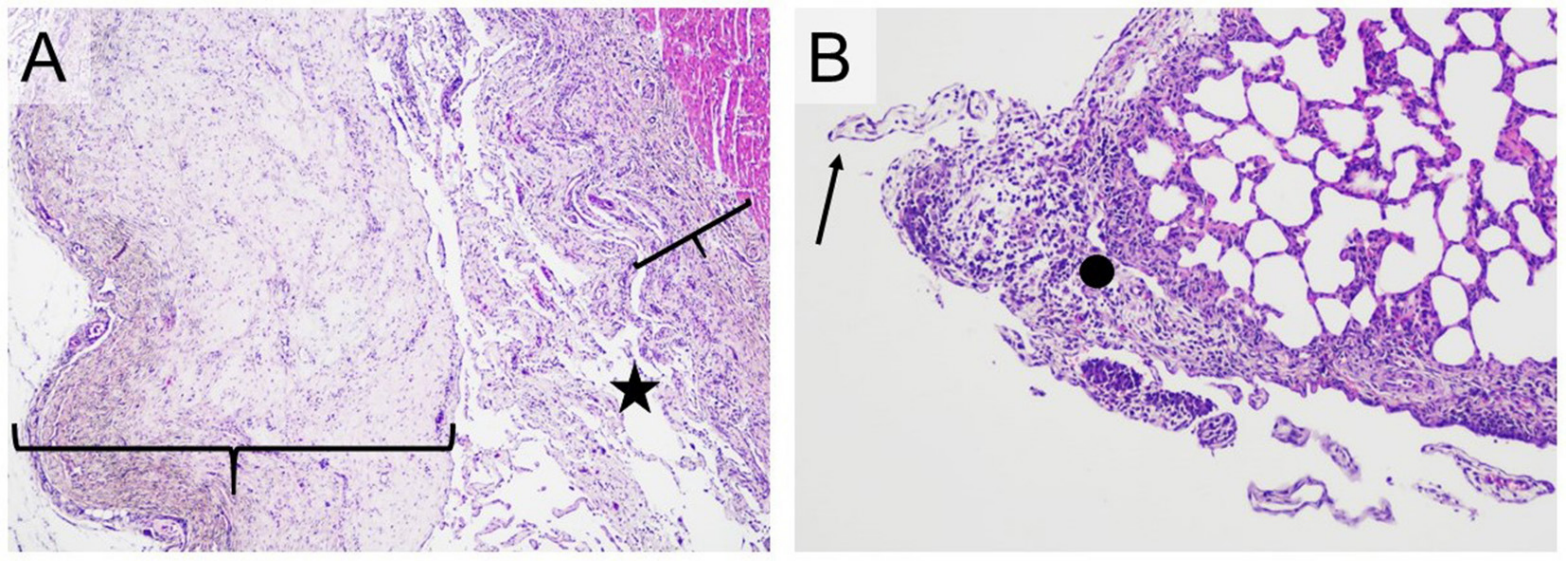
Typical histopathological changes in the serosa of *Mycoplasma hyorhinis* infected piglets. **(A)** A large number of filamentous projections consisting of connective tissue (star) and pronounced thickening of the pericardium and epicardium by proliferating connective tissue (braces; H&E, 40×). **(B)** Filamentous projections consisting of connective tissue (arrow) and focal, moderate thickening of the pleura by proliferating connective tissue with infiltration by mixed inflammatory cells (dot; H&E,100×).

The results of the histological examination are summarized in Supplementary Table 2. The main alterations were detected in the joints and in the serosa of parenchymal organs in the thoracic and peritoneal cavities. In the joints, lesions were detected in six out of six animals in Group IV-IV and four out of six animals in Group IV-IP. Joint lesions were evident in several cases only with histological examination. A total of 24 joints presented histological lesions (Supplementary Table 2), and most of these lesions (16 cases) were given a score of 3 (Figure 4; Supplementary Table 2). In the joints, inflammation was characterized by infiltration of mononuclear cells and, in more severe cases, hyperplasia of the synoviocytes (Supplementary Table 2). Lymphoid follicles formed around the blood vessels (four out of six animals in both Group IV-IV and IV-IP; Figure 5A) and fibrin exudates in the joint cavity were observed in several infected animals (Figure 5B; Supplementary Table 2). One animal in Group IV-IP presented acute-subacute erosive synovitis associated with acute hemorrhages and frequent occurrence of fibrin exudates, blood cells, and neutrophil granulocytes in the joint cavity (Figure 5B). Lesions of the serosal membranes (pleura, pericardium, and peritoneum) were characterized by the presence of filamentous projections consisting of connective tissue and by different severity of serosal thickening caused by proliferating connective tissue (Figures 4, 6; Supplementary Table 2). Alterations of the pleura were detected in three out of six pigs in Group IV-IV and four out of six pigs in Group IV-IP (Figures 4, 6B). However, lesions of the epi- and pericardium were detected in three out of six animals in Group IV-IV and two out of six animals in Group IV-IP (Figures 4, 6A). Finally, lesions of the peritoneum were presented in the capsule of the liver (one out of six animals in Group IV-IV) and the spleen (one animal in both challenge groups). Multinucleated giant cells in the joints in two cases and in the pleura in one of those cases were detected in the animals from Group IV-IV (Supplementary Tables 2, 10). Moreover, in Group IV-IV, one animal showed mild-to-moderate acute rhinitis, and in another case, acute ulcerative conjunctivitis was detected (Supplementary Tables 2, 10). No lesions were detected in the other organs. Combined macroscopic and histopathologic lesion scores with further details of the histopathologic lesions are given in Supplementary Table 10.

Scores of histological lesions of affected organs are shown in Figure 4. Based on the statistical analysis, scores of joints and total scores differed significantly between groups. In both cases, significant differences were detected between Group IV-IV and the control group (*p* = 0.04 regarding joint lesions, *p* = 0.04 regarding total score; Supplementary Data 1).

###  Serology

**Figure 7:**
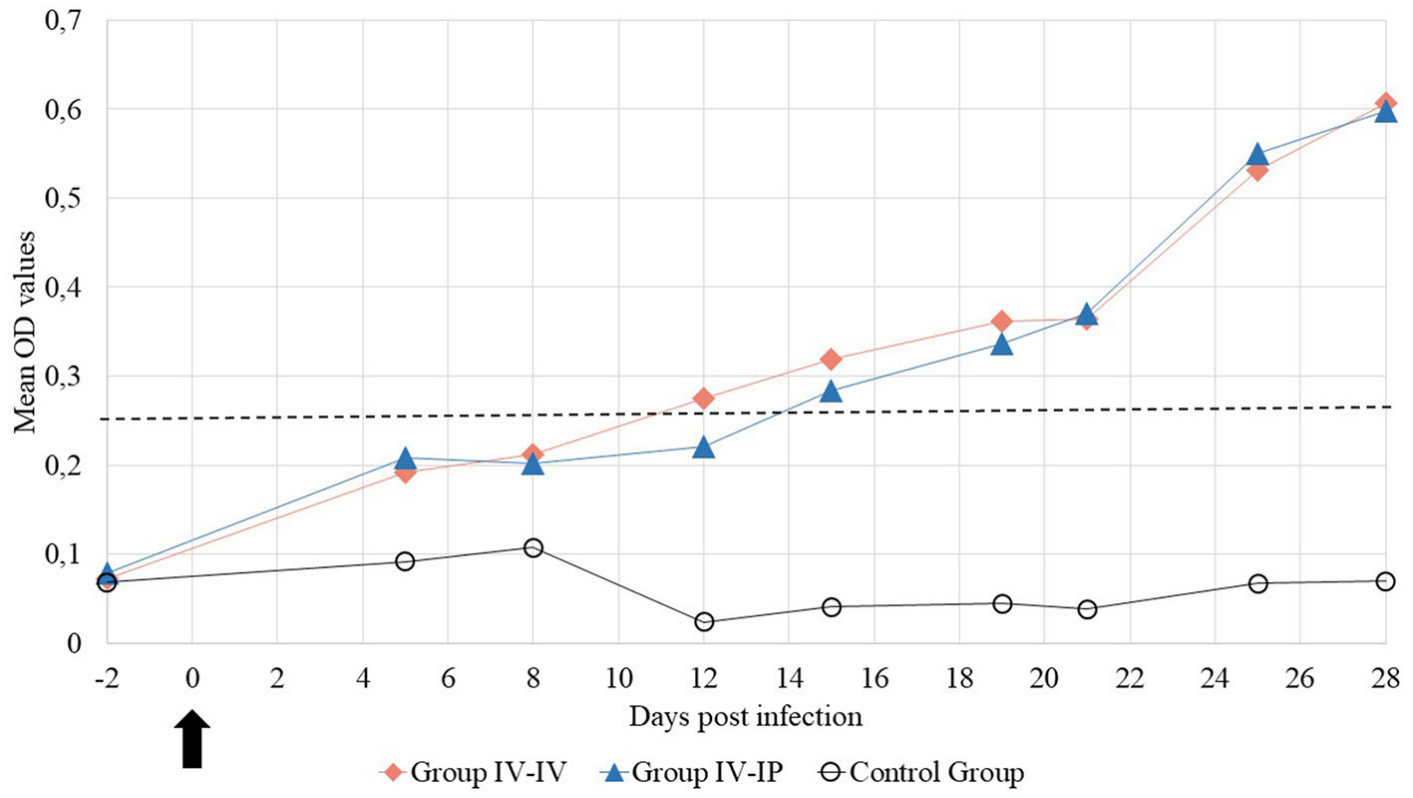
Mean optical density (OD) values of the blood samples during the study.

All animals were serologically negative for *M. hyorhinis* at the beginning of the study. The positive serological response appeared in Group IV-IP on 5 DPI, and the OD value of one out of six pigs was higher than the set threshold. The positive serological response was recorded in Group IV-IV at the next sampling on 8 DPI. By 28 DPI, all challenged animals were ELISA-positive (Supplementary Table 11). The mean OD values of the groups throughout the study are demonstrated in Figure 7. Significant differences in OD of 28 DPI were detected between the control group and Group IV-IV (*p* < 0.01) and the control group and Group IV-IP (*p* < 0.01; Supplementary Data 1). Animals from the control group remained negative throughout the study.

## Discussion

Based on available literature data, the single dose intranasal or intratracheal inoculation with *M. hyorhinis* is not suitable to establish a proper challenge model as these routes are usually only suitable to induce one aspect of the infection, mostly polyserositis and lung lesions with little or no serological conversion after infection (Lin et al., 2006; Gomes Neto et al., 2014; Lee et al., 2018; Fourour et al., 2019; Wei et al., 2020). Similarly, intranasal inoculation combined with tonsillar swabbing resulted in low serological conversion with no clinical signs or macroscopic lesions (Merodio et al., 2021). Time of challenge should not have an impact on the results of previous experiments as all studies used pigs at a receptive age (infected mostly at 6 weeks of age (Lin et al., 2006; Gomes Neto et al., 2014; Lee et al., 2018; Fourour et al., 2019; Merodio et al., 2021), or at 10 weeks of age (Wei et al., 2020). Our study plan was based on the study of Martinson et al. (2018a), where one-dose intranasal, intravenous, and intraperitoneal inoculations were compared to two- or three-dose inoculations with combined challenge routes in 7-week-old animals. The results of this study also confirmed that a single dose challenge is not sufficient to induce all typical lesions, with the mildest clinical signs observed in the intranasally infected group. On the other hand, in the intravenously infected group, the rate of pigs with pericarditis and pleuritis was similar to or higher than in the groups with combined challenge routes. The authors suggested the combination of intravenous, intraperitoneal, and intranasal routes on 3 consecutive days to induce both polyserositis and polyarthritis (Martinson et al., 2018a; Wang et al., 2022).

Typical lesions of *M. hyorhinis* infection are considered to be polyserositis and polyarthritis, while the involvement of *M. hyorhinis* in the etiology of lung lesions is still under discussion. Based on literature data, the role of *M. hyorhinis* as a secondary pathogen cannot be neglected in lung lesions as the presence of this pathogen induces more severe lesions in combination with porcine circovirus 2 and *M. hyopneumoniae* (Lee et al., 2016; Luehrs et al., 2017). However, in the challenge studies when both serositis (pericarditis, peritonitis, and pleuritis) and arthritis were induced, no lesions in the lungs were observed (Martinson et al., 2017, 2018a,b; Wang et al., 2022).

In the present study, two challenge routes were compared by using the same virulent clinical isolate. The double dose IV challenge (which was not mentioned in previous publications) produced equal involvement of joints as the mix of IV-IP route (arthritis of at least one joint was detected in 6/6 animals in both groups), which exceeded the rate of animals affected with arthritis in the previous study (single dose IV challenge resulted in arthritis in only 1/10 animal; Martinson et al., 2018a). The combination of the used infection routes (Group IV-IP) resulted in the earlier appearance of more pronounced clinical signs of arthritis like swollen joints and lameness. In the majority of animals affected with arthritis, the lesions indicated a subacute state of inflammation. Only a single animal in the IV-IP group presented acute inflammation in two joints. On the other hand, in the group which was challenged by the IV route on 2 consecutive days (Group IV-IV), the thoracic and peritoneal cavities were more commonly affected by serositis than in Group IV-IP, with equal involvement of the pericardial cavity in both groups. Most of the rest of the lesions appeared to be chronic in both infected groups. Chronic inflammation of the serosa presents as filamentous projections, serosal thickening, and adhesions between the serosal surfaces, which was detected both in the pericardium, peritoneum, and pleura of the affected animals. The low rate of re-isolation compared to PCR-positive samples also indicates the late phase of infection. Therefore, the reduction of the length of the study is suggested. Based on field observations and data from other challenge studies, the clinical signs gradually start to alleviate 2 weeks after the first clinical signs (Barden and Decker, 1971; Wang et al., 2022). Consequently, the length of the study should be determined based on the appearance of the first clinical signs (5 DPI here) and should be determined 14 days after (approximately 19 DPI in the present case). Genotyping of the re-isolates from the challenge revealed that the challenge strain was isolated from the affected organs.

Although the natural route of infection is not yet fully understood, the results of the presented and previous challenge models using the IV route indicate that the circulatory system has an important role in the systemic spread of *M. hyorhinis* (Martinson et al., 2018a). In the present study, both applied challenge routes included intravenous infection, and systemic spread of *M. hyorhinis* was obtained in both cases. Furthermore, despite inoculating directly the peritoneum in Group IV-IP, peritonitis was only detected in one animal by histopathology without macroscopic lesions, while in Group IV-IV macroscopic alterations and more pronounced histopathologic lesions of peritonitis were detected. Lesions of the pleura and pericardium were also more severe in Group IV-IV. Nevertheless, as with both challenge methods, the main lesions of *M. hyorhinis* infection were induced, and both models can be recommended for the future study of *M. hyorhinis* infection or vaccine efficacy studies. Considering the hypothesis of the natural spread of the pathogen via the circulatory or lymphatic system and the more severe pathological lesions in Group IV-IV, a two-dose intravenous challenge is recommended by the authors.

## Data availability statement

The original contributions presented in the study are included in the article/Supplementary material, further inquiries can be directed to the corresponding author.

## Ethics statement

The animal study was reviewed and approved by the National Scientific Ethical Committee on Animal Experimentation under reference number: PE/EA/746-7/2021.

## Author contributions

DF: investigation, formal analysis, and writing. ZN, NB, and LS: investigation. JF: supervision. AK and MT: conceptualization. ZK: editing and reviewing. MG: conceptualization, editing and reviewing, funding acquisition, and supervision. All authors contributed to the article and approved the submitted version.
