# Supplementary material for: Establishment of a Mycoplasma hyorhinis challenge model in 5-week-old piglets
Source: Front Microbiol. 2023 Aug 4;14:1209119. doi: 10.3389/fmicb.2023.1209119 (PMC10436309; doi:10.3389/fmicb.2023.1209119)
Supplement: Supplementary file 2 [file Data_Sheet_1.pdf]

Supplementary Material

Establishment of a *Mycoplasma hyorhinis* challenge model in five-week-old piglets

Dorottya Földi, Zsófia Eszter Nagy, Nikolett Belecz, Levente Szeredi, József Földi, Anna Kollár, Miklós Tenk, Zsuzsa Kreizinger, Miklós Gyuranecz*

*** Correspondence:** Corresponding Author: **m.gyuranecz@gmail.com**

1 Supplementary Data

1.1 Supplementary Data 1: Results of the statistical analysis.

a) Statistical analysis of daily weight gain

	Number of animals	Mean	SD
Group IV-IV	6	223	86.90
Group IV-IP	6	170	41.00
Control	4	350	99.00

Shapiro-Wilk normality test

Null hypothesis: the distribution of the data is not significantly different form normal distribution.

W=0.89, p-value=0.06

one-way ANOVA

	DF	Sum Sq	Mean Sq	F-value	Pr (>F)
Group	2	79067	39533	6.80	**<0.01**
Residuals	13	75533	5810		

Tukey multiple comparisons of means

Comparison	diff	lwr	upr	adjusted p-value
Group IV-IP-Group IV-IV	-53.33	-169.54	-62.87	0.47
Control-Group IV-IV	126.67	-3.25	256.58	**0.05**
Control-Group IV-IP	180.00	50.08	309.92	**<0.01**

b) Statistical analysis of gross pathological scores of joint lesions

	Number of animals	Median	IQR
Group IV-IV	6	1.50	3.25
Group IV-IP	6	3.00	1.50
Control	4	0.00	0.00

Kruskal-Wallis test	
chi-squared	9.03
DF	2
p-value	**0.01**

Dunn-test

Comparison	Z-value	unadjusted p-value	adjusted p-value
Group IV-IV-Group IV-IP	-0.25	0.80	1.00
Group IV-IV- Control	2.57	0.01	**0.03**
Group IV-IP- Control	2.79	0.01	**0.02**

c) Statistical analysis of gross pathological scores of serosa of pericardium, pleura and peritoneum

	Number of animals	Median	IQR
Group IV-IV	6	0.50	1.75
Group IV-IP	6	0.00	1.50
Control	4	0.00	0.00

Kruskal-Wallis test	
chi-squared	2.54
DF	2
p-value	0.28

d) Statistical analysis of total gross pathological scores

	Number of animals	Median	IQR
Group IV-IV	6	3.00	3.50
Group IV-IP	6	3.00	3.00
Control	4	0.00	0.00

Kruskal-Wallis test	
chi-squared	8.76
DF	2
p-value	**0.01**

Dunn-test			
Comparison	Z-value	unadjusted p-value	adjusted p-value
Group IV-IV-Group IV-IP	0.06	0.95	1.00
Group IV-IV- Control	2.67	0.01	**0.02**
Group IV-IP- Control	2.62	0.01	**0.03**

e) Statistical analysis of joint histology scores

	Number of animals	Median	IQR
Group IV-IV	6	3.50	1.00
Group IV-IP	6	3.50	7.75
Control	4	0.00	0.00

Kruskal-Wallis test	
chi-squared	6.48
DF	2
p-value	**0.04**

Dunn-test			
Comparison	Z-value	unadjusted p-value	adjusted p-value
Group IV-IV-Group IV-IP	0.50	0.62	1.00
Group IV-IV- Control	2.46	0.01	**0.04**
Group IV-IP- Control	2.01	0.04	0.12

f) Statistical analysis of the histology scores of serosa of pericardium, pleura and peritoneum

	Number of animals	Median	IQR
Group IV-IV	6	3.00	1.50
Group IV-IP	6	2.00	3.50
Control	4	0.00	0.00

Kruskal-Wallis test	
chi-squared	5.56
DF	2
p-value	0.06

g) Statistical analysis of total histology scores

	Number of animals	Median	IQR
Group IV-IV	6	6.00	3.00
Group IV-IP	6	5.50	12.80
Control	4	0.00	0.00

Kruskal-Wallis test	
chi-squared	6.64
DF	2
p-value	**0.04**

Dunn-test			
Comparison	Z-value	unadjusted p-value	adjusted p-value
Group IV-IV-Group IV-IP	0.62	0.53	1.00
Group IV-IV- Control	2.51	0.01	**0.04**
Group IV-IP- Control	1.95	0.51	0.15

h) Statistical analysis of ELISA S/P% from the last sampling point

	Number of animals	Mean	SD
Group IV-IV	6	0.61	0.17
Group IV-IP	6	0.60	0.21
Control	4	0.07	0.05

Shapiro-Wilk normality test

Null hypothesis: the distribution of the data is not significantly different form normal distribution.

W=0.94, p-value=0.38

one-way ANOVA

	DF	Sum Sq	Mean Sq	F-value	Pr (>F)
Group	2	0.853	0.427	14.85	**<0.01**
Residuals	13	0.374	0.023		

Tukey multiple comparisons of means

Comparison	diff	lwr	upr	adjusted p-value
Group IV-IP-Control Group	0.53	0.24	0.82	**<0.01**
Group IV-IV-Control Group	0.54	0.25	0.83	**<0.01**
Group IV-IV-Group IV-IP	0.01	-0.25	0.27	**0.99**

1.2 Supplementary data 2: Aligned, concatenated sequences of the multi-locus sequence typing of the challenge strain and the re-isolates.

Provided in separate file.

2 Supplementary Figures and Tables

2.1 Supplementary Figures

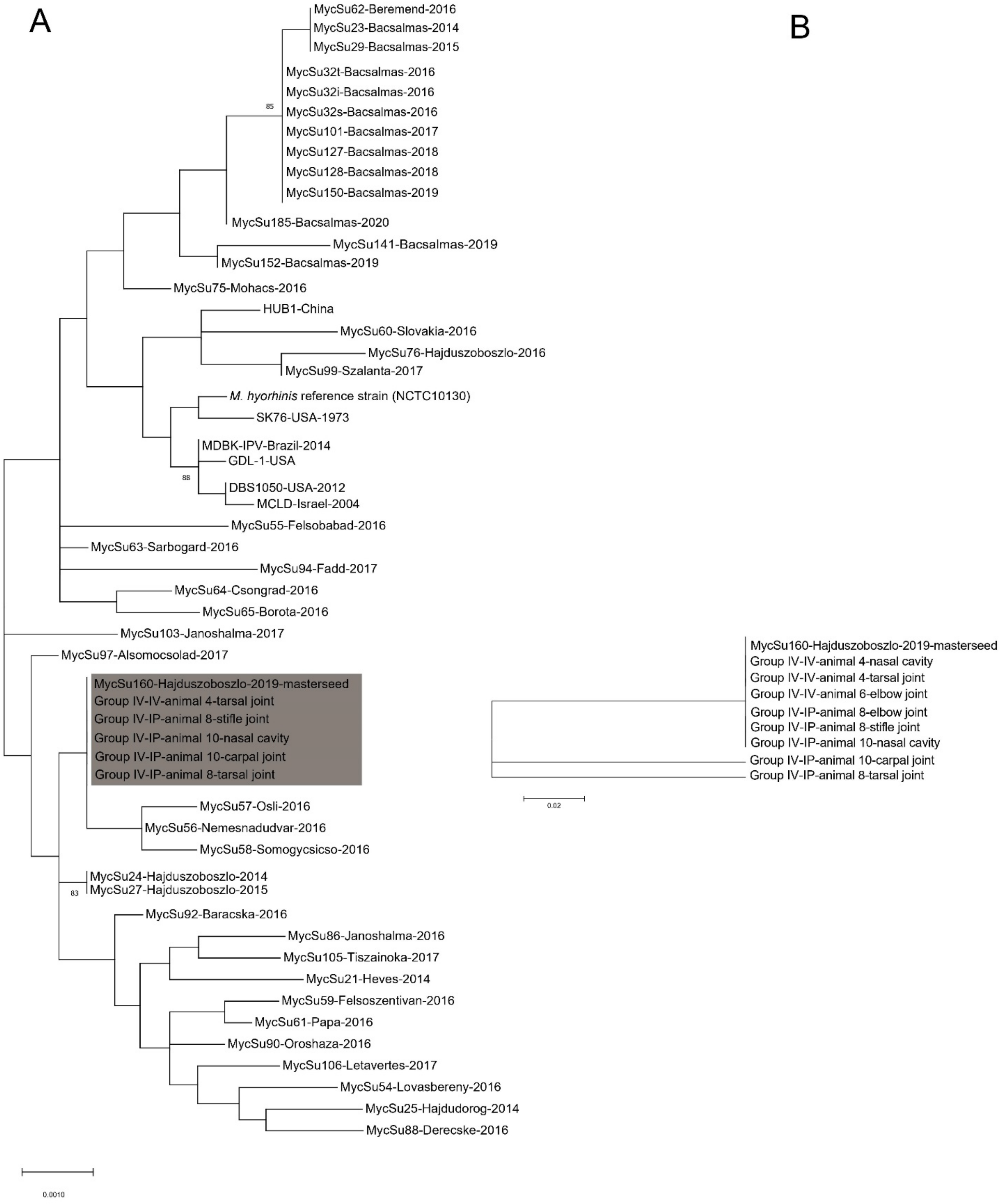
A
MycSu62-Beremend-2016
MycSu23-Bacsalmas-2014
MycSu29-Bacsalmas-2015
MycSu32t-Bacsalmas-2016
MycSu32i-Bacsalmas-2016
85
MycSu32s-Bacsalmas-2016
MycSu101-Bacsalmas-2017
MycSu127-Bacsalmas-2018
MycSu128-Bacsalmas-2018
MycSu150-Bacsalmas-2019
MycSu185-Bacsalmas-2020
MycSu141-Bacsalmas-2019
MycSu152-Bacsalmas-2019
MycSu75-Mohacs-2016
HUB1-China
MycSu60-Slovakia-2016
MycSu76-Hajduszoboszlo-2016
MycSu99-Szalanta-2017
M. hyorhinis reference strain (NCTC10130)
SK76-USA-1973
MDBK-IPV-Brazil-2014
GDL-1-USA
88
DBS1050-USA-2012
MCLD-Israel-2004
MycSu55-Felsobabad-2016
MycSu63-Sarbogard-2016
MycSu94-Fadd-2017
MycSu64-Csongrad-2016
MycSu65-Borota-2016
MycSu103-Janoshalma-2017
MycSu97-Alsomocsolad-2017
MycSu160-Hajduszoboszlo-2019-masterseed
Group IV-IV-animal 4-tarsal joint
Group IV-IP-animal 8-stifle joint
Group IV-IP-animal 10-nasal cavity
Group IV-IP-animal 10-carpal joint
Group IV-IP-animal 8-tarsal joint
MycSu57-Osli-2016
MycSu56-Nemesnadudvar-2016
MycSu58-Somogycsicso-2016
MycSu24-Hajduszoboszlo-2014
83
MycSu27-Hajduszoboszlo-2015
MycSu92-Baracska-2016
MycSu86-Janoshalma-2016
MycSu105-Tiszainoka-2017
MycSu21-Heves-2014
MycSu59-Felsoszentivan-2016
MycSu61-Papa-2016
MycSu90-Oroshaza-2016
MycSu106-Letavertes-2017
MycSu54-Lovasbereny-2016
MycSu25-Hajdudorog-2014
MycSu88-Derecske-2016
0.0010
B
MycSu160-Hajduszoboszlo-2019-masterseed
Group IV-IV-animal 4-nasal cavity
Group IV-IV-animal 4-tarsal joint
Group IV-IV-animal 6-elbow joint
Group IV-IP-animal 8-elbow joint
Group IV-IP-animal 8-stifle joint
Group IV-IP-animal 10-nasal cavity
Group IV-IP-animal 10-carpal joint
Group IV-IP-animal 8-tarsal joint
0.02

Supplementary Figure 1. Dendrograms of multi-locus sequence typing (MLST, A) and multiple-locus variable-number tandem-repeat analysis (MLVA, B). A. The MLST tree was constructed by using the Maximum Likelihood method, Hasegawa-Kishino-Yano model in the MegaX software (Kumar, Stecher, Li, Knyaz, & Tamura, 2018). Gene fragments from lepA, rpoB, rpoC, gltX, valS and uvrA were used, with 1000 bootstraps (only bootstrap values >70% are presented). B. Resolution of the identical sequence type of the isolates from the present study was carried out with MLVA based on Mhr205, Mhr396, Mhr438, Mhr441, Mhr442 and Mhr444 alleles. The tree was constructed by the Neighbour-Joining method.

Isolates from this study are highlighted in light grey on the MLST tree. MLST sequences are available in Supplementary Data 2, and tandem-repeat numbers of the isolates from this study can be found in Supplementary Table 8. Data from the other isolates was previously published in Földi et al., 2020.

2.2 Supplementary tables (provided in a separate file)
